# Evaluating large language models in specialized myopia knowledge and clinical reasoning

**DOI:** 10.3389/fmed.2026.1886498

**Published:** 2026-08-06

**Authors:** Zhilin Li, Na Xin, Tai Wang, Xiaodong Li, Xiyu Jia

**Affiliations:** 1 Guangzhou Medical University, Guangzhou, China; 2 The Third Affiliated Hospital (The Affiliated Luohu Hospital) of Shenzhen University, Shenzhen University, Shenzhen, China; 3 Shenzhen Eye Hospital, Shenzhen Eye Medical Center, Southern Medical University, Shenzhen, China; 4 Department of Ophthalmology, The Second People's Hospital of Chengdu Pidu District, Chengdu, China; 5 The First Affiliated Hospital of Guizhou University of Traditional Chinese Medicine, Guiyang, China

**Keywords:** clinical reasoning, large language models, myopia, ophthalmology, patient consultation

## Abstract

**Background:**

Myopia is a major global public health concern, and the growing use of LLMs for health consultation makes the accuracy of their myopia-related responses increasingly important. Existing ophthalmic LLM studies have largely focused on image-based applications and mixed ocular diseases, with limited evaluation of myopia-specific reasoning. Yet myopia-related inquiries span both breadth and depth, from specialized differential considerations to common issues in prevention, control, and public education. This study aimed to assess mainstream LLMs across multiple levels of myopia-related knowledge and clinical reasoning.

**Methods:**

This comparative evaluation study assessed six large language models: GPT-5.3 Instant, DeepSeek-V3.2, Gemini 3.1 Pro Preview, Claude Opus 4.7, Qwen3.6-Plus, and Doubao-Seed-2.0-Pro. The assessment covered four clinically relevant domains of myopia care and included hierarchical case-based scenarios spanning core judgment, preferred management, and follow-up. Objective items were submitted in independent new chat sessions, repeated three times, and tested without additional medically enhancing prompts. For the open-ended case tasks, responses from the six models and eight junior physicians were independently scored by two senior ophthalmology experts.

**Results:**

ChatGPT showed the highest accuracy in standardized judgment tasks, case-based clinical tasks, and external validation multiple-choice questions, achieving 92.33% ± 1.15%, 85.00% ± 1.67%, and 88.00% ± 1.00%, respectively, with DeepSeek ranking second. In case-based scenarios, model performance was strongest for core judgment but declined notably for preferred management decisions. In the open-ended case assessment, ChatGPT, DeepSeek, Claude, and Gemini achieved higher expert-rated scores than junior clinicians, whereas Doubao and Qwen showed lower overall performance. Across the four clinical domains, performance differences widened in tasks requiring applied management and referral reasoning.

**Conclusions:**

Current LLMs demonstrated substantial capability in myopia-related knowledge assessment and text-based clinical reasoning. For patients and the public, these models may provide convenient access to relatively professional responses to common myopia-related questions. Although several models achieved higher expert-rated scores than junior clinicians in standardized written cases, this advantage was limited to text-based assessment and should not be extrapolated to real-world clinical performance. Given the complexity of specialist judgment and individual variation, model-generated responses should serve as informative references for consultation, health education, and preliminary decision support, not substitutes for formal clinical advice or physician-led individualized management.

## 1 Introduction

Myopia, along with its associated complications, constitutes a significant global public health concern (1), has received increasing attention in recent years (2). Myopia is a common cause of vision loss. Research predicts that by 2050, 4.758 billion people will suffer from myopia (approximately 49.8% of the world's total population), including 938 million people with high myopia (3). With the increase in the incidence of high myopia, the complications it causes, such as retinal detachment, macular degeneration, glaucoma, posterior scleral staphyloma, etc., will also increase (4). Increasing levels of myopia carry significant clinical and economic implications, with more people at risk of the sight-threatening complications associated with high myopia (5). In addition, the genetic nature of myopia also poses obstacles to myopia prevention and control. Therefore, early prevention of myopia may be the best option for myopia prevention and control (6). Equally important is how to help the general public and grassroots medical personnel make professional judgments regarding myopia. However, prior ophthalmic evaluations have predominantly focused on other ocular diseases, leaving myopia comparatively underexplored (7).

In recent years, generative artificial intelligence, especially large language models (LLMs), has attracted increasing attention for its potential applications in medical information support and clinical decision support. Leveraging natural language understanding and generation capabilities, LLMs can provide health education information to patients and parents through interactive question-and-answer sessions, and can also provide clinicians with diagnostic and treatment approaches, supplementary knowledge, and decision-making references. In the field of ophthalmology, previous research has begun to explore the applications of LLMs in model building, disease diagnosis, and professional knowledge question answering (8). However, existing evidence still suggests that LLMs' performance in professional medical scenarios is not always consistent: the accuracy of their answers may vary depending on the task type and knowledge domain, and may fail to reflect the latest medical advancements in a timely manner when limited by the timeliness of training data; their reasoning and response abilities in complex or rare clinical situations remain uncertain (9). Furthermore, LLMs can also create “illusions,” generating information that appears plausible but is in fact erroneous or lacks basis, thus posing a challenge to the safety of medical applications (10). Therefore, in myopia-related applications, in addition to focusing on whether the model can answer questions, it is more necessary to systematically evaluate whether its performance is stable under different task formats, different clinical content, and different application scenarios, and further examine whether its capabilities can reach or approach the level of a junior physician. This study follows the TITAN 2025 recommendations on the transparent and responsible use of artificial intelligence technology in research (11).

**Figure 1:**
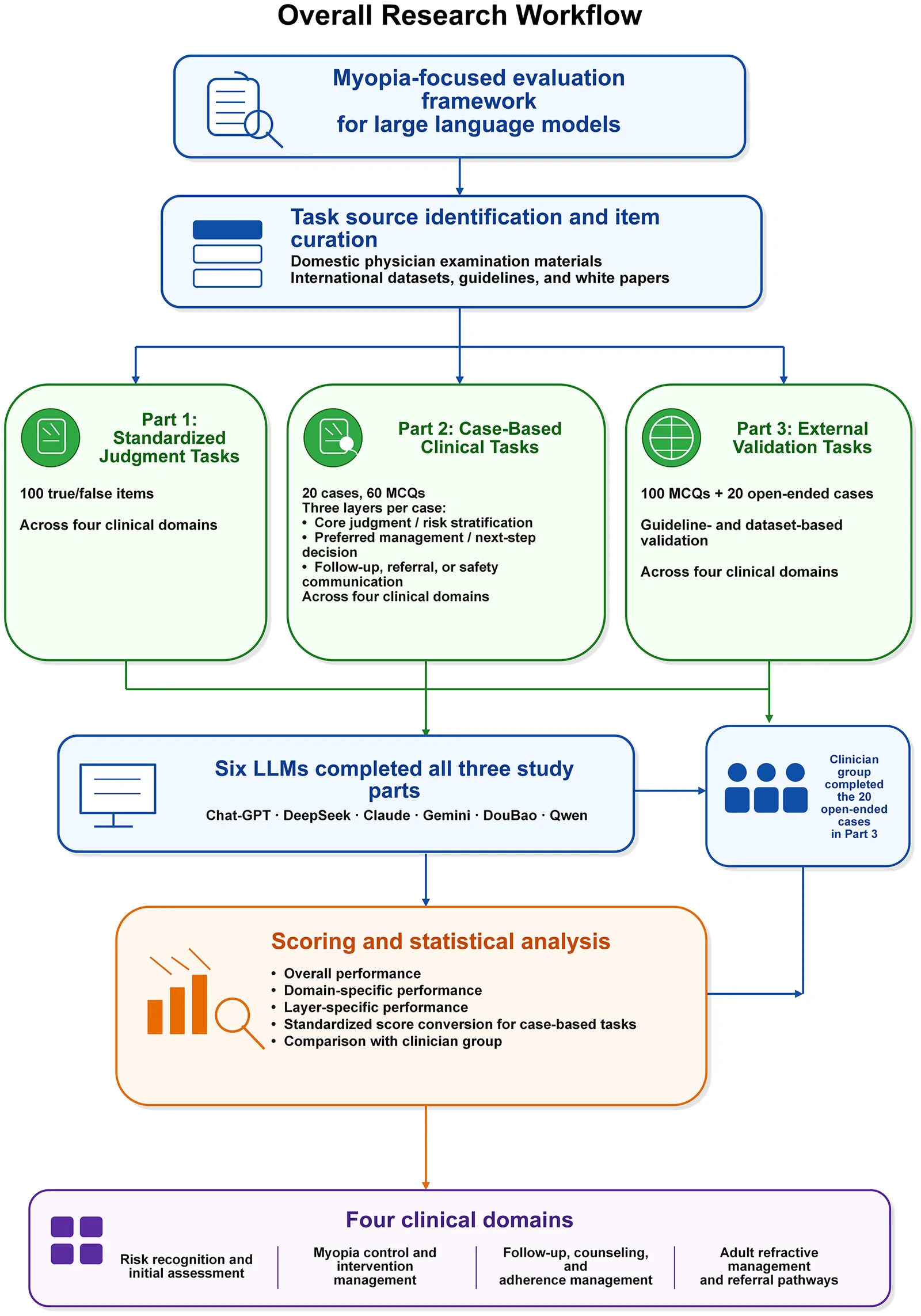
Overall study design and evaluation workflow. The study evaluated six large language models using a multi-level myopia assessment framework comprising objective knowledge tasks, case-based reasoning tasks, external validation, and open-ended case evaluation with clinician comparison.

Although large language models (LLMs) have shown promising performance across a range of medical tasks (12), and preliminary applications have been reported in myopia-related health education (13), and orthokeratology care (14). Their capacity to address professional clinical issues related to myopia has not been systematically evaluated. To address this gap, we developed a myopia-focused, three-part evaluation framework to examine whether LLMs can maintain robust performance across different task formats and clinical scenarios. Part 1 consisted of 100 standardized true-or-false judgment items. Part 2 included 20 case-based clinical scenarios, with three multiple-choice questions generated for each case to assess different levels of clinical reasoning, yielding 60 items in total. The questions in Parts 1 and 2 were derived from domestic physician licensing and intermediate professional qualification examinations. Part 3 served as an external validation module, with items developed from international guidelines (15), white papers (16), and publicly available datasets (17); it comprised 100 multiple-choice questions and 20 open-ended case tasks. This framework was designed to evaluate the breadth, stability, and practical clinical relevance of LLM performance in myopia-related decision-making, and to further explore whether their capabilities approach those of junior clinicians in selected applied scenarios. The overall study design and technical workflow are shown in Figure 1 and Table 1.

**Table 1:** Research design and composition of the study.

Research section	Source	Question types	Total number of questions	Task structure	Risk recognition and initial assessment	Myopia control and intervention management	Follow-up, counseling, and adherence management	Adult refractive management and referral pathways	Primary evaluation objectives
Part 1: standardized true/false assessment	National Licensed Physician Examination, Intermediate-Level Ophthalmology Professional Qualification Examination	True/false questions	100	One question, one judgment	25	25	25	25	Assess the model's mastery of core knowledge related to myopia, risk identification, and basic judgment
Part 2: case-based clinical task assessment	Clinical case materials from the National Licensed Physician Examination and the Intermediate-Level Ophthalmology Professional Qualification Examination, restructured	20 case studies, totaling 60 multiple-choice questions	60	Three questions per case: core judgment/risk stratification; preferred management/next steps; key points for follow-up, referral, or safety communication	15	15	15	15	Evaluate the model's judgment, management, and safety capabilities in clinical myopia practice scenarios
Part 3: independent validation set assessment	Public datasets, international guidelines, expert consensus documents, and white papers	Part 3A: multiple-choice questions	100	One question, one judgment	25	25	25	25	Validate the model's knowledge consistency and judgment stability across different data sources and regulatory frameworks
		Part 3B: case studies	20	One case, one task	5	5	5	5	Validate the model's clinical transferability and application stability across case scenarios from different sources

## 2 Materials and methods

### 2.1 Study design and evaluation framework

This study was designed to systematically evaluate the performance of six large language models (LLMs) in myopia-related knowledge assessment and clinical reasoning. The evaluated models included ChatGPT, DeepSeek, Gemini, Claude, Qwen, and Doubao, using the latest publicly available versions as of April 20, 2026: GPT-5.3 Instant, DeepSeek-V3.2, Gemini 3.1 Pro Preview, Claude Opus 4.7, Qwen3.6-Plus, and Doubao-Seed-2.0-Pro.

The evaluation framework comprised three parts. Part 1 consisted of 100 standardized true-or-false judgment items. Part 2 included 20 case-based clinical scenarios, with three multiple-choice questions generated for each case to assess different levels of clinical reasoning, yielding 60 items in total. The questions in Parts 1 and 2 were derived from domestic physician licensing and intermediate professional qualification examinations. Items in Parts 1 and 2 were translated from Chinese into English and reviewed by a medical English translation specialist and an ophthalmologist to ensure linguistic accuracy, terminological consistency, and clinical equivalence. Part 3 served as an external validation module, with items developed from international guidelines, white papers, and publicly available datasets; it comprised 100 multiple-choice questions and 20 open-ended case tasks (18, 19). Across all three parts, items were included if they assessed core ophthalmic knowledge or clinically relevant decision-making, had a clearly defined reference answer, contained sufficient information for independent interpretation, and were supported by authoritative sources. Duplicated, highly similar, ambiguous, outdated, image-dependent, excessively region-specific, or unverifiable items were excluded.

Reference answers for Parts 1 and 2 were based on official or published examination answers and cross-checked against current ophthalmology guidelines and authoritative textbooks. Reference answers for Part 3 were derived from the corresponding international guidelines, expert consensus statements, white papers, or public datasets. All selected items and reference answers were independently reviewed by two ophthalmologists, and disagreements were resolved through discussion and consensus.

To minimize contextual carryover and reduce potential bias, each question was submitted in an independent new chat session without customized prompts or additional contextual instructions. Each item was tested three times for each model to assess response stability and reproducibility.

### 2.2 Human comparison and expert scoring

For the 20 open-ended case tasks in Part 3, the responses generated by the LLMs were compared with those provided by eight junior physicians. All responses were independently evaluated by two senior ophthalmology experts with advanced professional titles using a predefined scoring rubric. The rubric assessed the quality of the responses according to clinically relevant criteria, including accuracy, completeness, appropriateness of reasoning, and practical applicability. Discrepancies between expert ratings were resolved through discussion and consensus. Consensus scores were used for the primary analysis because they represented the final adjudicated expert assessment after resolution of scoring discrepancies, whereas the independent pre-consensus scores were used only to assess inter-rater reliability.

### 2.3 Statistical analysis

For the objective items in Parts 1, 2, and the multiple-choice component of Part 3, model performance was assessed using accuracy rates and error rates. Repeated testing results were summarized to evaluate response consistency. Because the same fixed item set was tested repeatedly, the three runs were interpreted as repeated model outputs for assessing response stochasticity and stability, rather than as independently sampled item populations. For the open-ended case tasks, rubric-based scores were used to compare the performance of LLMs and junior physicians. Statistical analyses were conducted using GraphPad Prism 10. Continuous variables were summarized as mean ± standard deviation or mean ± standard error, as appropriate. Differences among multiple groups were analyzed using one-way analysis of variance with *post hoc* multiple-comparison testing when applicable. Variance homogeneity was assessed using the Brown–Forsythe test; ordinary one-way ANOVA with Tukey's multiple-comparison test was used when homogeneity was satisfied, whereas Welch ANOVA with Games–Howell testing was used when it was violated. As a sensitivity analysis for the objective modules, paired item-level logistic regression was performed using response correctness as the binary outcome, with model as the main explanatory variable and item identity and run included to account for the paired item structure and repeated testing. A two-sided *p* value < 0.05 was considered statistically significant.

## 3 Results

### 3.1 Overall performance across the study framework

**Figure 2:**
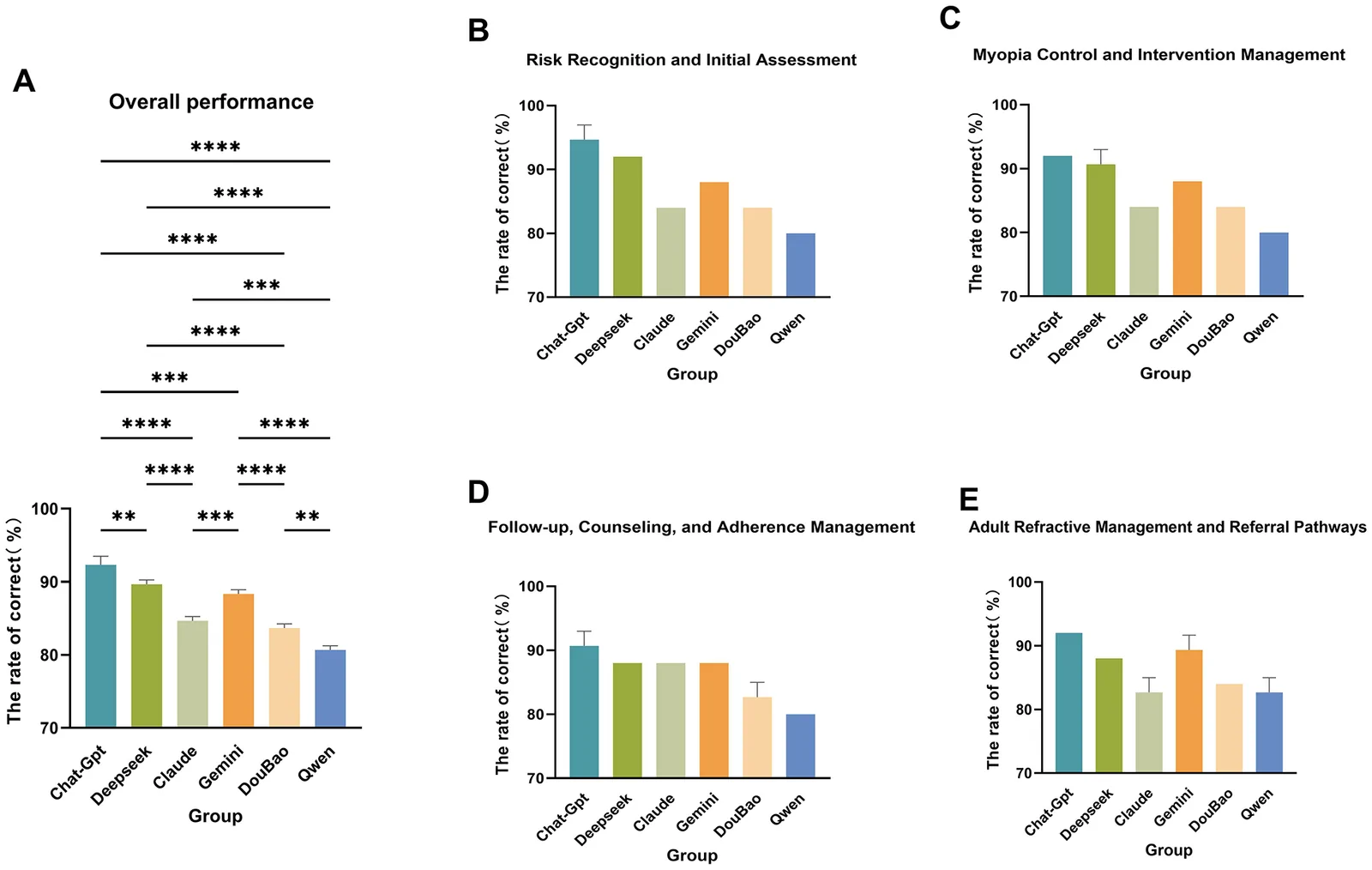
Overall and domain-specific performance of six large language models in standardized judgment tasks. **(A)** Overall accuracy of the six models in Part 1 standardized judgment tasks. **(B–E)** Domain-specific accuracy across risk recognition and initial assessment, myopia control and intervention management, follow-up, counseling, and adherence management, and adult refractive management and referral pathways. ** indicates *P* ≤ 0.01, *** indicates *P* ≤ 0.001, and **** indicates *P* ≤ 0.0001.

**Figure 3:**
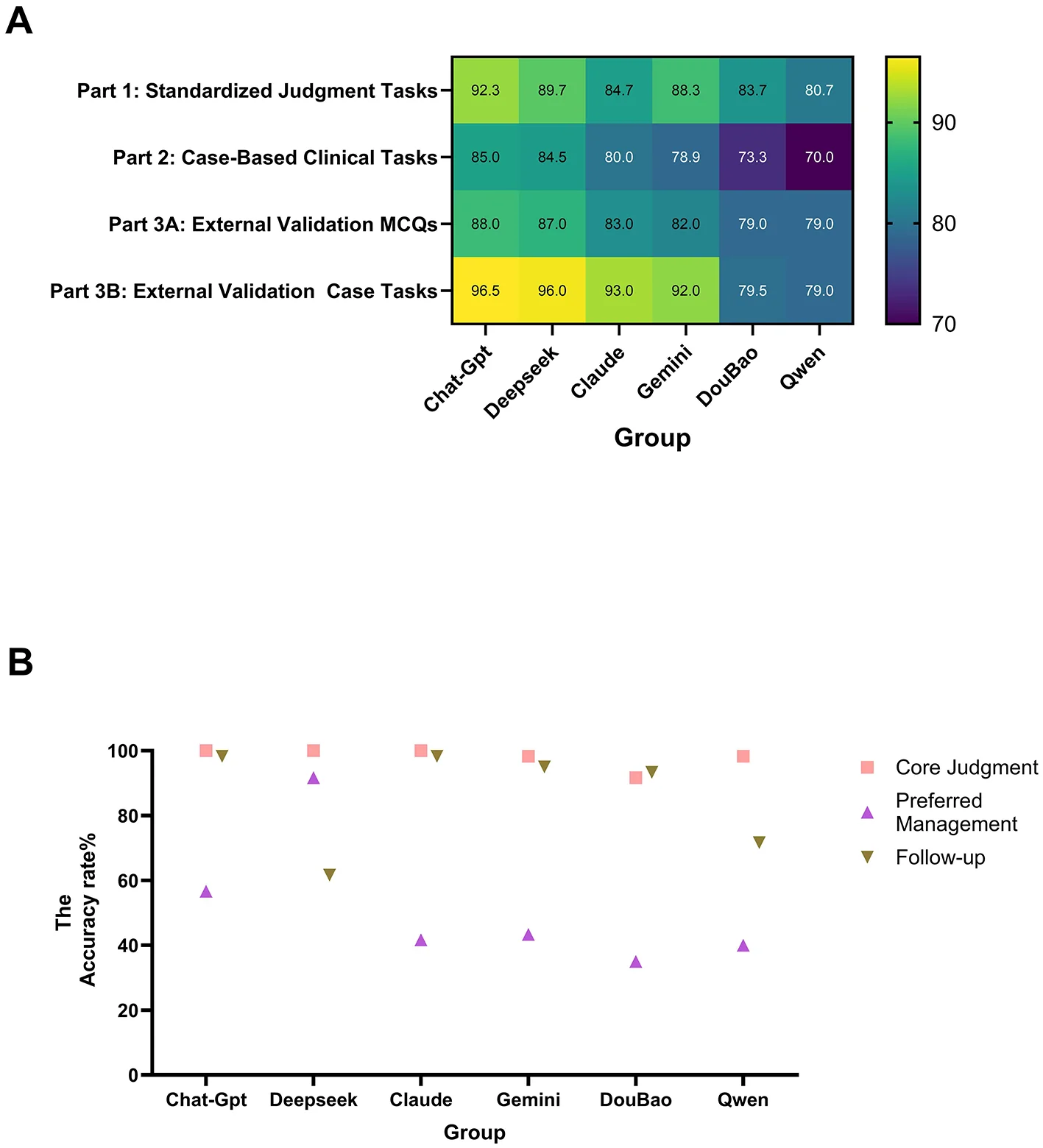
Overall and task-level performance of six large language models in myopia-related assessment. **(A)** Overall performance of the six models across standardized judgment tasks, case-based clinical tasks, external validation multiple-choice questions, and external validation case tasks. **(B)** Accuracy of the six models across the three hierarchical components of the case-based clinical tasks: core judgment, preferred management, and follow-up.

Across the three-part evaluation framework, the six LLMs showed clear differences in myopia-related knowledge assessment and clinical reasoning performance. In Part 1, ChatGPT achieved the highest overall accuracy in standardized judgment tasks, followed by DeepSeek and Gemini, as shown in Figure 2. A similar ranking pattern was observed in Part 2 case-based clinical tasks and Part 3A external validation multiple-choice questions. Run-level group differences were observed in Part 1, Part 2, and Part 3A. Variance homogeneity was assessed using the Brown–Forsythe test and was not violated; therefore, ordinary one-way ANOVA with Tukey's multiple-comparison testing was used. Significant model differences were observed in Part 1, Part 2, and Part 3A [Part 1: *F*_(5, 12)_ = 111.38, *P* < 0.001; Part 2: *F*_(5, 12)_ = 34.77, *P* < 0.001; Part 3A: *F*_(5, 12)_ = 44.40, *P* < 0.001]. These ANOVA results were interpreted as exploratory run-level comparisons because the three repeated runs were generated from the same fixed item set. The paired item-level logistic regression sensitivity analysis showed a pattern consistent with the run-level ANOVA results across Parts 1, 2, and 3A, and the main interpretation of model performance differences remained unchanged. The overall performance of each model across the three objective evaluation modules is summarized in Table 2 and Figure 3A.

**Table 2:** Overall performance of six models across the three study parts.

Research Structure	Metrics	Chat-Gpt	DeepSeek	Claude	Gemini	DouBao	Qwen
Part 1: standardized judgment tasks	Overall accuracy (%)	92.33 ± 1.15	89.67 ± 0.58	84.67 ± 0.58	88.33 ± 0.58	83.67 ± 0.58	80.67± 0.58
Part 2: case-based clinical tasks	Overall accuracy by question type (%)	85.00 ± 1.67	84.44 ± 2.55	80.00 ± 1.67	78.89 ± 0.96	73.33 ± 1.67	70.00 ± 1.67
Part 3A: external validation multiple-choice tasks	Overall accuracy for multiple-choice questions (%)	88.00 ± 1.00	87.00 ± 1.00	83.00 ± 1.00	82.00 ± 1.00	79.00 ± 1.00	79.00 ± 1.00
Part 3B: external validation open-ended case tasks	Overall performance on case-based questions (%)	96.5 ± 5.9	96.0 ± 5.0	93.0 ± 7.3	92.0 ± 12.4	79.5 ± 20.1	79.0 ± 17.1

To enable direct comparison across different task formats, case-based scores were converted to percentages using the formula: observed score/maximum score × 100%. For case-based tasks, mean ± SD was calculated from normalized per-case scores, rather than from repeated total scores as in the objective tasks.

### 3.2 Performance on standardized judgment and case-based clinical tasks

**Figure 4:**
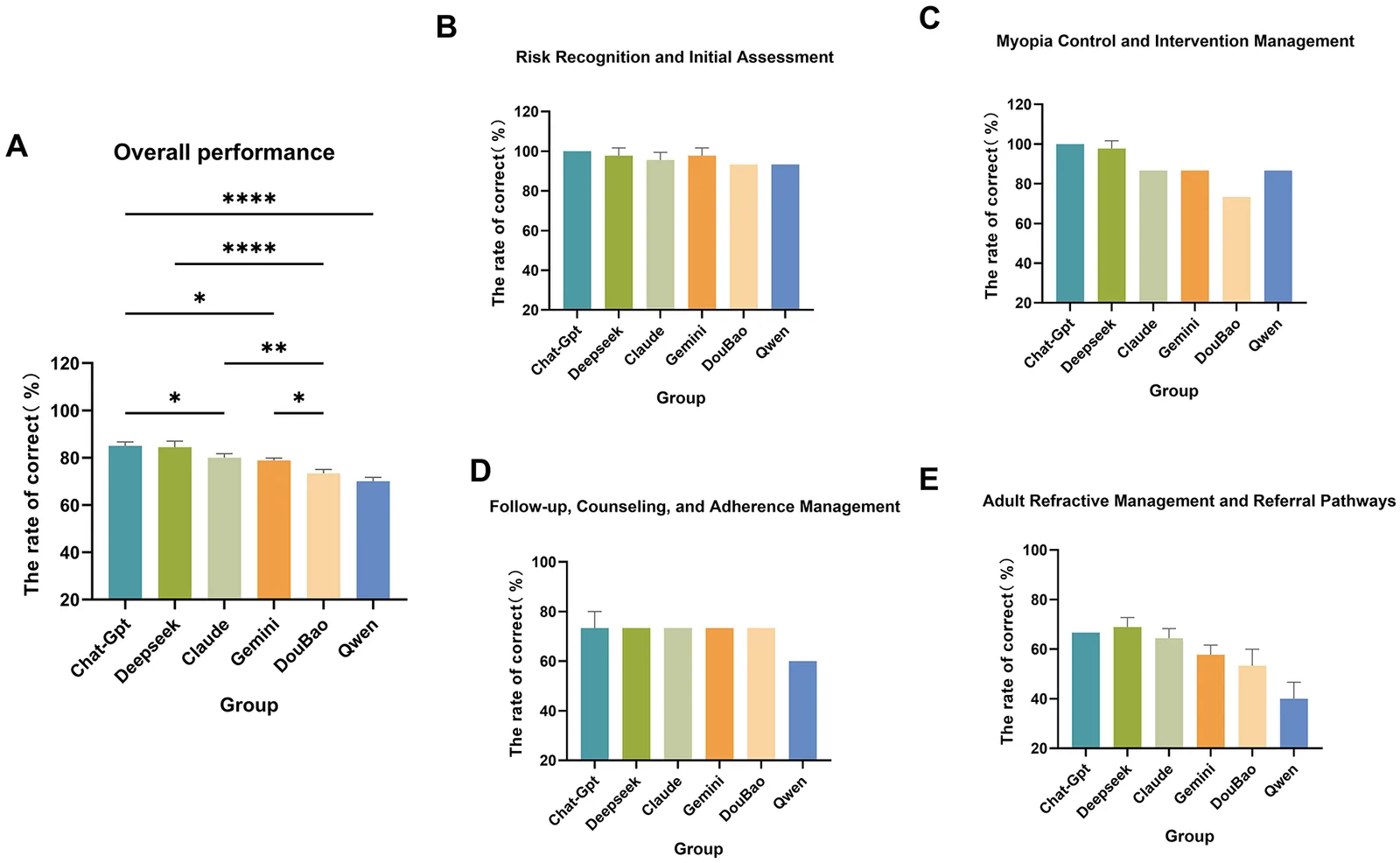
Overall and domain-specific performance of six large language models in case-based clinical tasks. **(A)** Overall accuracy of the six models in Part 2 case-based clinical tasks. **(B–E)** Domain-specific accuracy across risk recognition and initial assessment, myopia control and intervention management, follow-up, counseling, and adherence management, and adult refractive management and referral pathways. * indicates *P* ≤ 0.05, ** indicates *P* ≤ 0.01, *** indicates *P* ≤ 0.001, and **** indicates *P* ≤ 0.0001.

In Part 1, ChatGPT obtained the highest accuracy for standardized true-or-false judgment items at 92.33% ± 1.15%, followed by DeepSeek at 89.67% ± 0.58% and Gemini at 88.33% ± 0.58%. Qwen showed the lowest overall accuracy at 80.67% ± 0.58%. In Part 2, ChatGPT again achieved the highest overall accuracy in case-based clinical tasks at 85.00% ± 1.67%, closely followed by DeepSeek at 84.44% ± 2.55%. Each of the 20 cases contained three single-best-answer questions. Claude, Gemini, DouBao, and Qwen achieved accuracies of 80.00% ± 1.67%, 78.89% ± 0.96%, 73.33% ± 1.67%, and 70.00% ± 1.67%, respectively, as shown in Figure 2. When the three-tiered case structure in Part 2 was examined separately, all models performed strongly on core judgment items, whereas substantially lower performance was observed for preferred management questions, particularly among Claude, Gemini, DouBao, and Qwen. Follow-up questions showed comparatively higher accuracy than preferred management questions for most models. These findings are detailed in Tables 2, 3. A task-level analysis of Part 2 further showed that all models performed better on core judgment items, whereas accuracy declined substantially for preferred management questions, as illustrated in Figures 3B, 4.

**Table 3:** Results table for the three-tiered case study in Part 2.

Section	Chat-Gpt	DeepSeek	Claude	Gemini	DouBao	Qwen
Core judgment	100	100	100	98.33	91.67	98.33
Preferred management	56.67	91.67	41.67	43.33	35	40
Follow-up	98.33	61.67	98.33	95	93.33	71.67

### 3.3 External validation performance

**Figure 5:**
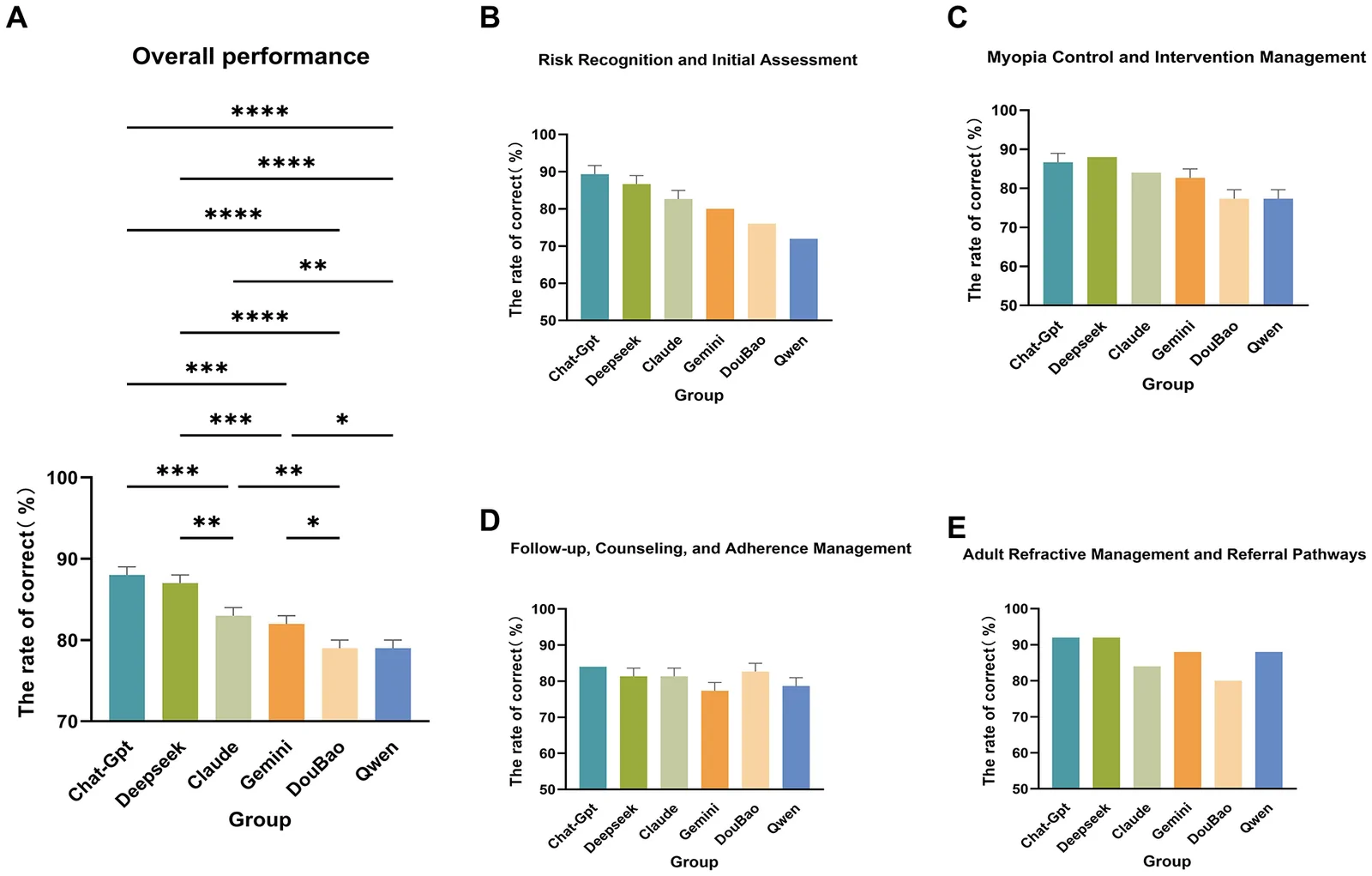
Overall and domain-specific performance of six large language models in external validation multiple-choice tasks. **(A)** Overall accuracy of the six models in Part 3A external validation multiple-choice tasks. **(B–E)** Domain-specific accuracy across risk recognition and initial assessment, myopia control and intervention management, follow-up, counseling, and adherence management, and adult refractive management and referral pathways. * indicates *P* ≤ 0.05, ** indicates *P* ≤ 0.01, *** indicates *P* ≤ 0.001, and **** indicates *P* ≤ 0.0001.

In Part 3A, which assessed external validation using multiple-choice questions developed from international guidelines, white papers, expert consensus documents, and public datasets, ChatGPT achieved the highest overall accuracy at 88.00% ± 1.00%, followed by DeepSeek at 87.00% ± 1.00%. Claude and Gemini reached 83.00% ± 1.00% and 82.00% ± 1.00%, respectively, while DouBao and Qwen both achieved 79.00% ± 1.00%. Across the four predefined clinical domains, ChatGPT and DeepSeek generally maintained leading performance, although domain-specific variation was observed. For example, ChatGPT and DeepSeek both reached 92.00% in adult refractive management and referral pathways, whereas performance was comparatively lower in follow-up, counseling, and adherence management. The detailed domain-stratified results are presented in Table 4 and Figure 5.

**Table 4:** Model performance stratified by four clinical domains across the three study sub-groups.

Research structure	Sections	Chat-Gpt	DeepSeek	Claude	Gemini	DouBao	Qwen
Part 1: standardized judgment tasks	Risk recognition and initial assessment	94.67 ± 2.31	92.00 ± 0.00	84.00 ± 0.00	88.00 ± 0.00	84.00 ± 0.00	80.00 ± 0.00
	Myopia control and intervention management	92.00 ± 0.00	90.67 ± 2.31	84.00 ± 0.00	88.00 ± 0.00	84.00 ± 0.00	80.00 ± 0.00
	Follow-up, counseling, and adherence management	90.67 ± 2.31	88.00 ± 0.00	88.00 ± 0.00	88.00 ± 0.00	82.67 ± 2.31	80.00 ± 0.00
	Adult refractive management and referral pathways	92.00 ± 0.00	88.00 ± 0.00	82.67 ± 2.31	89.33 ± 2.31	84.00 ± 0.00	82.67 ± 2.31
Part 2: case-based clinical tasks	Risk recognition and initial assessment	100.00 ± 0.00	97.78 ± 3.85	95.56 ± 3.85	97.78 ± 3.85	93.33 ± 0.00	93.33 ± 0.00
	Myopia control and intervention management	100.00 ± 0.00	97.78 ± 3.85	86.67 ± 0.00	86.67 ± 0.00	73.33 ± 0.00	86.67 ± 0.00
	Follow-up, counseling, and adherence management	73.33 ± 6.67	73.33 ± 0.00	73.33 ± 0.00	73.33 ± 0.00	73.33 ± 0.00	60.00 ± 0.00
	Adult refractive management and referral pathways	66.67 ± 0.00	68.89 ± 3.85	64.44 ± 3.85	57.78 ± 3.85	53.33 ± 6.67	40.00 ± 6.67
Part 3A: external validation multiple-choice tasks	Risk recognition and initial assessment	89.33 ± 2.31	86.67 ± 2.31	82.67 ± 2.31	80.00 ± 0.00	76.00 ± 0.00	72.00 ± 0.00
	Myopia control and intervention management	86.67 ± 2.31	88.00 ± 0.00	84.00 ± 0.00	82.67 ± 2.31	77.33 ± 2.31	77.33 ± 2.31
	Follow-up, counseling, and adherence management	84.00 ± 0.00	81.33 ± 2.31	81.33 ± 2.31	77.33 ± 2.31	82.67 ± 2.31	78.67 ± 2.31
	Adult refractive management and referral pathways	92.00 ± 0.00	92.00 ± 0.00	84.00 ± 0.00	88.00 ± 0.00	80.00 ± 0.00	88.00 ± 0.00
Part 3B: external validation open-ended case tasks	Risk recognition and initial assessment	98.0 ± 4.5	98.0 ± 4.5	92.0 ± 8.4	82.0 ± 17.9	58.0 ± 14.8	68.0 ± 22.8
	Myopia control and intervention management	94.0 ± 5.5	94.0 ± 5.5	92.0 ± 8.4	96.0 ± 5.5	88.0 ± 8.4	80.0 ± 0.0
	Follow-up, counseling, and adherence management	94.0 ± 8.9	96.0 ± 5.5	88.0 ± 4.5	98.0 ± 4.5	92.0 ± 13.0	90.0 ± 14.1
	Adult refractive management and referral pathways	100.0 ± 0.0	96.0 ± 5.5	100.0 ± 0.0	92.0 ± 13.0	80.0 ± 24.5	78.0 ± 19.2

All figures in the table are expressed as percentages; marks for clinical case questions have been converted to “actual marks/maximum marks × 100%” for the purposes of comparison. For case-based tasks, mean ± SD was calculated from normalized per-case scores, rather than from repeated total scores as in the objective tasks.

### 3.4 Open-ended clinical case tasks and comparison with junior clinicians

**Figure 6:**
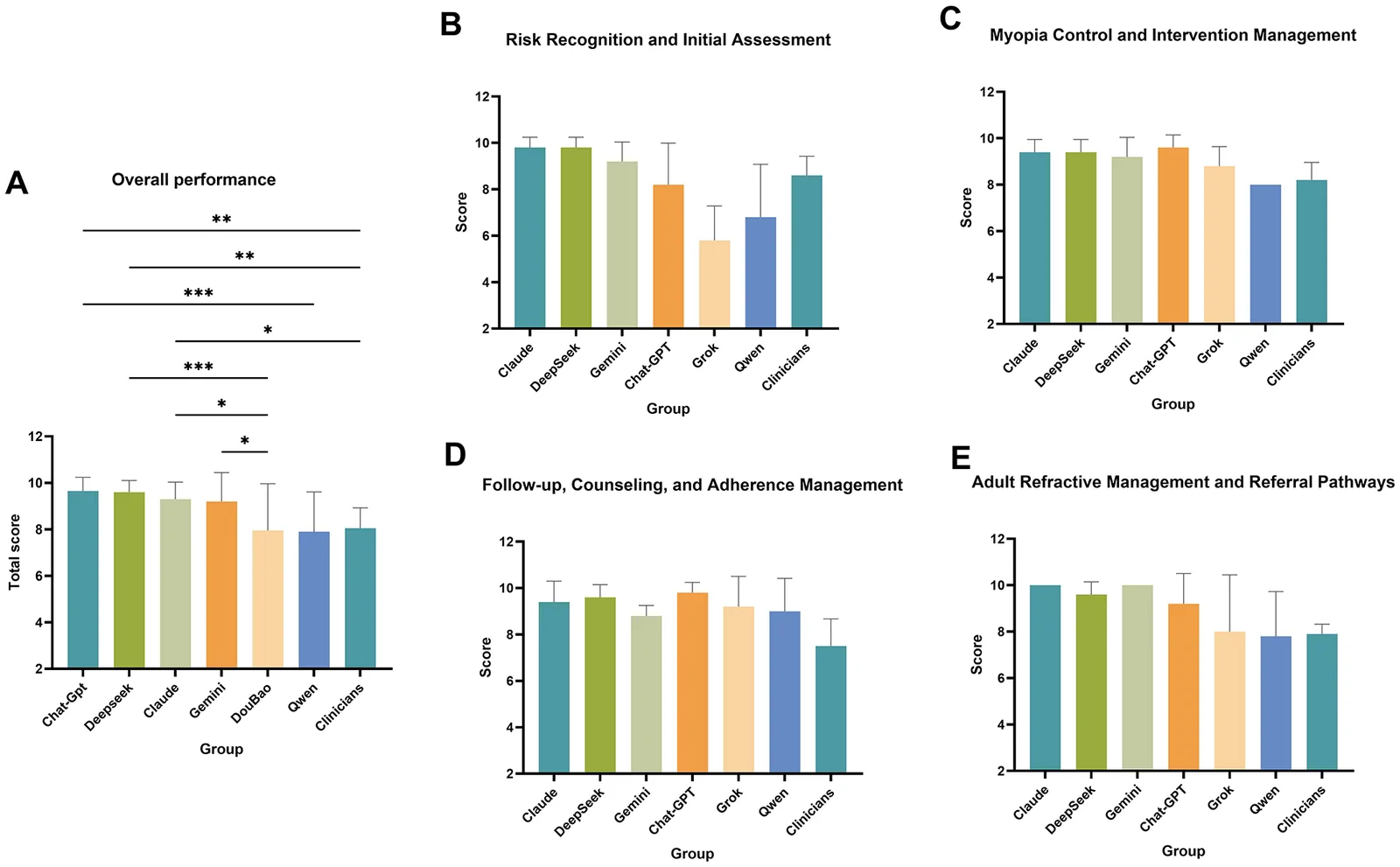
Overall and domain-specific performance of large language models and junior clinicians in external validation open-ended case tasks. **(A)** Overall normalized performance scores of the six models and junior clinicians in Part 3B external validation open-ended case tasks. **(B–E)** Domain-specific normalized scores across risk recognition and initial assessment, myopia control and intervention management, follow-up, counseling, and adherence management, and adult refractive management and referral pathways. * indicates *P* ≤ 0.05, ** indicates *P* ≤ 0.01, *** indicates *P* ≤ 0.001, and **** indicates *P* ≤ 0.0001.

In Part 3B, model performance on 20 open-ended external validation case tasks was assessed using normalized consensus scores from two senior ophthalmologists. Inter-rater reliability, calculated from their independent pre-consensus scores, was good to excellent for model-generated responses [ICC(2,1) = 0.886]. ChatGPT, DeepSeek, Claude, and Gemini achieved mean normalized scores of 96.5% ± 5.9%, 96.0% ± 5.0%, 93.0% ± 7.3%, and 92.0% ± 12.4%, respectively, whereas DouBao and Qwen obtained lower scores of 79.5% ± 20.1% and 79.0% ± 17.1%. Because variance homogeneity was not satisfied according to the Brown–Forsythe test, group differences were evaluated using Welch ANOVA, which demonstrated a significant overall difference among the six LLMs and junior clinicians [*F*_(6, 58.07)_ = 12.23, *P* < 0.001]. Games–Howell *post hoc* testing showed that ChatGPT, DeepSeek, Claude, and Gemini scored significantly higher than junior clinicians, whereas DouBao and Qwen did not show significant differences from the clinician group. Median and interquartile range values for each group are reported descriptively in Table 5 and Figure 6, while the inferential comparison was based on the Prism-derived mean score data.

**Table 5:** Performance on the open-ended case tasks in the independent validation set (Part 3) across the four clinical domains [median (IQR)].

Group	Chat-Gpt	Deepseek	Claude	Gemini	DouBao	Qwen	Clinicians
Total score	10.0 (9.0–10.0)	10.0 (9.0–10.0)	9.0 (9.0–10.0)	10.0 (9.0–10.0)	8.0 (6.8–10.0)	8.0 (7.0–9.0)	9.5 (8.0–10.0)
Risk recognition and initial assessment	10.0 (10.0–10.0)	10.0 (10.0–10.0)	9.0 (9.0–10.0)	8.0 (7.0–10.0)	6.0 (5.0–6.0)	7.0 (7.0–8.0)	9.0 (8.0–9.5)
Myopia control and intervention management	9.0 (9.0–10.0)	9.0 (9.0–10.0)	9.0 (9.0–10.0)	10.0 (9.0–10.0)	9.0 (8.0–9.0)	8.0 (8.0–8.0)	9.0 (8.5–10.0)
Follow-up, counseling, and adherence management	10.0 (9.0–10.0)	10.0 (9.0–10.0)	9.0 (9.0–9.0)	10.0 (10.0–10.0)	10.0 (9.0–10.0)	10.0 (8.0–10.0)	8.0 (8.0–9.0)
Adult refractive management and referral pathways	10.0 (10.0–10.0)	10.0 (9.0–10.0)	10.0 (10.0–10.0)	10.0 (9.0–10.0)	8.0 (8.0–10.0)	8.0 (7.0–9.0)	7.5 (5.5–8.0)

Data are presented as median (interquartile range, IQR).

### 3.5 Domain-specific performance patterns

Across the four clinical domains, the strongest performance in Part 3B was generally observed among ChatGPT and DeepSeek, both of which maintained high scores in risk recognition, intervention management, follow-up counseling, and adult refractive management. Gemini showed particularly high scores in myopia control and intervention management as well as follow-up and adherence management, but lower performance in risk recognition and initial assessment. Claude achieved high performance in adult refractive management and referral pathways, while DouBao and Qwen showed more pronounced variability across domains, with their lowest scores appearing in risk recognition and initial assessment. A similar pattern of domain-dependent performance was also observed in Parts 1–3A, where follow-up-related and adult refractive management items tended to reveal greater divergence among models than basic risk recognition items. These stratified findings are summarized in Table 4 and Table 5.

## 4 Discussion

### 4.1 Principal findings

Previous studies on LLMs in myopia have largely focused on answering common patient questions (20), generating educational materials (13), or developing specialized consultation systems (21). More recent work has also begun to explore real-world pediatric myopia reasoning (22) or retrieval-augmented applications (23). In contrast, the present study provides a broader comparative assessment of mainstream LLMs across multiple levels of myopia-related competence, including standardized knowledge judgment, hierarchical case-based reasoning, external validation, and open-ended clinical transferability, with an additional comparison against junior clinicians. This design better reflects the diverse ways in which the public and frontline providers may engage with LLMs in myopia-related decision support.

This study systematically compared six widely used large language models across myopia-related knowledge assessment, case-based clinical reasoning, and external validation tasks. Clear intermodel differences were identified, with ChatGPT and DeepSeek showing the most consistently favorable performance across the principal evaluation modules. Although accuracy was generally high in standardized judgment tasks and external validation multiple-choice items, performance gaps widened in case-based scenarios requiring applied reasoning and management decisions. In open-ended external validation cases, ChatGPT, DeepSeek, Claude, and Gemini achieved significantly higher expert-rated scores than junior clinicians. These findings highlight the potential of advanced LLMs to support myopia-related clinical reasoning while underscoring the task-dependent nature of their performance.

### 4.2 Task- and domain-dependent differences in model performance

Model performance varied substantially across both task formats and clinical domains. In the case-based clinical assessment, all models performed strongly on core judgment items, whereas accuracy declined markedly for preferred management questions, indicating that identifying the central clinical issue is less demanding than selecting the most appropriate management strategy. DeepSeek appeared to adopt a more cautious approach in the “Preferred Management” tier, particularly by avoiding potentially unsafe interventions and favoring appropriate specialist evaluation. Performance on follow-up tasks partially recovered in several models, suggesting a relative strength in generating structured downstream recommendations compared with making immediate priority-based treatment decisions. A similar pattern emerged across the four predefined clinical domains: performance was generally more stable in risk recognition and initial assessment, while greater variability was observed in domains requiring broader contextual integration, particularly adult refractive management and referral pathways. These findings suggest that current LLMs are comparatively stronger in knowledge recognition and preliminary clinical judgment than in more complex management-oriented reasoning that requires nuanced prioritization and care-pathway selection.

### 4.3 External evaluation and clinical comparison

The external validation findings broaden the interpretation of the primary results. In Part 3A, models that performed better in the main evaluation generally retained strong accuracy on questions drawn from international guidelines, white papers, expert consensus documents, and public datasets, suggesting relatively stable knowledge performance across different evidence sources. Part 3B further showed that several models performed well on open-ended case tasks, indicating that their ability was not limited to selecting predefined answers but also extended to case-oriented reasoning and response formulation. These findings support the possible value of LLMs in myopia-related clinical support, especially when guideline-based knowledge needs to be applied to specific scenarios. However, good performance in text-based external validation should not be interpreted as readiness for direct clinical use, which requires further testing in more complex and realistic care settings.

The comparison with junior clinicians highlights the practical relevance of these findings. As the burden of myopia continues to grow, an increasing number of individuals may turn to LLMs for preliminary consultation and health information before seeking formal care, making the accuracy of model-generated judgments particularly important. In the open-ended case assessment, ChatGPT, DeepSeek, Claude, and Gemini achieved higher expert-rated scores than the clinician group, suggesting that some models can produce structured and clinically comprehensive responses in text-based scenarios. However, this comparison was based on predefined written cases rather than real-world patient encounters and should not be interpreted as evidence that LLMs can replace physicians. Instead, these findings support their potential role as adjunctive tools for patient guidance, health education, and selected forms of myopia-related clinical decision support.

### 4.4 Positioning within prior ophthalmic LLM research and relevance to myopia

Recent artificial intelligence research in ophthalmology has often emphasized image-centered applications, such as retinal disease recognition and systemic risk prediction from ocular imaging (24), while ophthalmic LLM studies have more commonly focused on mixed-disease diagnostic (25) and management tasks (26) across subspecialties rather than on myopia-specific reasoning. In contrast, myopia-related questions encountered by the public are frequently text based (27) and practical in nature, involving risk interpretation, prevention and control strategies, follow-up, and referral decisions. Against this background, the present study addresses a distinct and clinically relevant gap by evaluating LLMs from multiple perspectives and at multiple levels, including standardized knowledge judgment, case-based hierarchical reasoning, external validation, and open-ended clinical transferability. This design moves beyond conventional benchmarking and provides a more comprehensive assessment of whether current LLMs can support myopia-related information needs in a manner that is relevant not only to specialists and institutions, but also to broader public-facing health communication and early decision support.

### 4.5 Limitations and future directions

Several limitations should be considered. First, although this study incorporated examination-derived questions, international guidance documents, public datasets, and case-based tasks, these materials cannot fully reproduce the complexity of real clinical practice. Second, all evaluations were text based and did not include ophthalmic imaging, refraction reports, axial length trajectories, corneal topography, or other multimodal information that may influence myopia-related decisions (28). Third, model performance is inherently time sensitive because commercially available LLMs are updated rapidly, which may affect reproducibility across testing periods. Fourth, the clinician comparison was limited to junior physicians and expert-scored written case responses, and therefore should not be generalized to broader physician populations or direct clinical performance. Finally, despite the use of predefined scoring criteria, the assessment of open-ended responses inevitably retains some subjectivity. These limitations align with broader concerns in the current LLM literature regarding evaluation standardization, robustness, reliability, and clinical generalizability. Future studies should therefore incorporate more realistic care scenarios, multimodal information, larger and more diverse human comparator groups, and more detailed assessment of safety, hallucination risk, and longitudinal consistency before clinical deployment is considered.

## 5 Conclusions

In conclusion, LLMs demonstrated meaningful but heterogeneous performance across myopia-related knowledge assessment, clinical reasoning, and external validation tasks, with ChatGPT and DeepSeek showing the most consistently favorable results. These findings suggest that advanced LLMs may have practical value in supporting public-facing myopia consultation, health education, and preliminary decision assistance. However, their use should remain cautious and adjunctive, and further validation in more realistic, multimodal, and clinically integrated settings is warranted.

## Data Availability

The raw data supporting the conclusions of this article will be made available by the authors, without undue reservation.
