# Supplementary material for: Evaluating large language models in specialized myopia knowledge and clinical reasoning
Source: Front Med (Lausanne). 2026 Aug 6;13:1886498. doi: 10.3389/fmed.2026.1886498 (PMC13489900; doi:10.3389/fmed.2026.1886498)
Supplement: Supplementary file 1 [file Table_2.docx]

The following standardized, model-neutral prompt was used for all evaluated large language models. The same prompt structure was applied across models and runs. No model-specific instructions, examples, feedback, or additional contextual information were provided.

Standardized prompt template

You are asked to complete an ophthalmology-related assessment task. Please read each item carefully and answer strictly according to the question type and the required output format.

If the item is a multiple-choice question, select only one best answer from A, B, C, or D. Do not provide explanations, reasoning steps, comments, or additional information. Return only the final selected option in the required field.

If the item is a case-based question, provide a concise and clinically relevant response according to the following four predefined tasks:

Core judgment: summarize the main clinical judgment, risk stratification, or key problem in the case.

Preferred management: provide the most appropriate initial or next-step management recommendation.

Follow-up and referral: indicate whether further examination, referral, or follow-up is needed, and specify the recommended timing where applicable.

Safety communication: identify the key safety concern and the main point that should be communicated to the patient or caregivers.

For case-based questions, answers should be based only on the information provided in the case. Do not copy the case stem verbatim. Keep each task response concise, professional, and clinically focused. If the available information is insufficient, indicate what additional information or examination is needed while still completing all four tasks.

Please follow the predefined output fields exactly. Do not add new fields, remove fields, provide a preface, summary, disclaimer, markdown formatting, or explanatory text outside the required output.

Output requirements

For multiple-choice questions, use the following fields:

question_id

module

question_type

model_name

run_id

run_date

language

prompt_version

selected_option

For case-based questions, use the following fields:

case_id

module

question_type

model_name

run_id

run_date

language

prompt_version

task1_core_judgment

task2_first_management

task3_followup_referral

task4_safety_communication

The output should be returned in the predefined JSON format only.
