# Supplementary material for: Evaluating large language models in specialized myopia knowledge and clinical reasoning
Source: Front Med (Lausanne). 2026 Aug 6;13:1886498. doi: 10.3389/fmed.2026.1886498 (PMC13489900; doi:10.3389/fmed.2026.1886498)
Supplement: Supplementary file 2 [file Table_3.docx]

# Scope of scoring

**The rubric was applied only to the 20 open-ended case tasks in Part 3B.** Parts 1, 2, and 3A were objective items scored against predefined reference answers. Each Part 3B response was evaluated according to four predefined clinical tasks: core clinical judgment, preferred management, follow-up/referral planning, and safety communication. The total score for each case ranged from 0 to 10 points.

# Scoring domains and weights

| **Scoring domain** | **Maximum score** | **Weight in total score** | **Rationale for weighting** |
| --- | --- | --- | --- |
| Core clinical judgment and risk stratification | 0–3 points | 30% | This domain captures whether the response identifies the main clinical problem, risk level, and red-flag features. It was assigned a higher weight because incorrect initial judgment can lead to inappropriate downstream management. |
| Preferred management | 0–3 points | 30% | This domain assesses whether the response recommends the most appropriate first-line or next-step management. It was assigned a higher weight because management choice is directly linked to clinical utility and patient safety. |
| Follow-up/referral planning | 0–2 points | 20% | This domain evaluates the appropriateness of additional examinations, referral decisions, and follow-up timing. It was weighted separately because timely reassessment and referral are essential in high-risk myopia-related scenarios. |
| Safety communication | 0–2 points | 20% | This domain assesses whether the response communicates key risks and counseling points to patients or caregivers. Although scored with fewer points than judgment and management, it is essential for safe clinical implementation. |

| **Domain** | **Score range** | **Scoring criteria** | **Examples of deduction or loss of credit** |
| --- | --- | --- | --- |
| Task 1: Core clinical judgment | 0–3 | 3 = core judgment is fully correct, with clear identification of the major diagnosis/problem and risk stratification; 2 = generally correct but incomplete; 1 = partially relevant but with important deviation; 0 = incorrect judgment or omission of key risk. | Misclassifying a high-risk case as low risk; treating a potentially pathological or asymmetric presentation as ordinary myopia; failing to recognize warning signs such as unilateral progression, new floaters/flashes, or high-myopia retinal risk. |
| Task 2: Preferred management | 0–3 | 3 = management recommendation is appropriate for the clinical stage and consistent with standardized care; 2 = generally reasonable but insufficiently specific; 1 = vague or suboptimal recommendation; 0 = clearly inappropriate management. | Recommending observation when active intervention or urgent evaluation is indicated; delaying necessary retinal/corneal specialist assessment; recommending an inappropriate surgical or procedural intervention. |
| Task 3: Follow-up/referral planning | 0–2 | 2 = follow-up timing, additional examination, and/or referral recommendation is appropriate and specific; 1 = follow-up/referral is mentioned but incomplete or nonspecific; 0 = omitted or clearly inappropriate. | Failure to recommend prompt fundus or specialist evaluation when red-flag symptoms are present; giving an excessively long follow-up interval for a high-risk case; omitting necessary baseline or structural assessment. |
| Task 4: Safety communication | 0–2 | 2 = key safety concern and patient/caregiver counseling point are clearly identified; 1 = safety issue is mentioned but incomplete; 0 = no meaningful safety warning or misleading reassurance. | Downplaying red-flag signs; giving overconfident assurances such as “absolutely safe” or “definitely suitable for surgery”; failing to explain why delayed care or poor adherence may be risky. |

# Detailed 10-point scoring rubric

# Total score and safety-error flag

**Total score.** The total score was calculated as the sum of the four task scores: Task 1 + Task 2 + Task 3 + Task 4. The maximum total score per case was 10 points. Higher scores indicate better clinical accuracy, completeness, appropriateness, and practical applicability.

**Serious safety error.** In addition to the total score, serious safety errors were recorded as a separate binary flag and were not directly incorporated into the 10-point total. This separate flag was used because a response may achieve a relatively high total score while still containing a clinically important unsafe recommendation.

| **Serious safety error criterion** | **Examples** |
| --- | --- |
| Failure to recommend urgent or timely referral when clearly indicated | New floaters/flashes in a highly myopic patient without prompt fundus evaluation or retinal referral. |
| Incorrectly reassuring a high-risk condition as ordinary or low-risk myopia | Marked unilateral progression or high-risk axial elongation treated as normal variation without further assessment. |
| Definitive surgical recommendation despite insufficient information or contraindications | Recommending refractive surgery without adequate corneal, ocular surface, or stability assessment. |
| Potentially harmful or unsafe management advice | Additional corneal laser ablation in suspected post-LASIK ectasia or other structurally unsafe scenarios. |

# Scoring procedure

**•** Each open-ended response was scored using the same four-domain rubric.

**•** Case-specific reference-answer key points were used to guide scoring, but credit was awarded for clinically equivalent wording rather than exact phrase matching.

**•** Responses were scored for clinical correctness, completeness, appropriateness of reasoning, practical applicability, and patient-safety implications.

**•** Final analytic scores were based on consensus scoring. If independent pre-consensus scores are used for reliability analysis, inter-rater reliability should be calculated from those independent scores before consensus, while the consensus scores can be retained for the main outcome analysis.

# Interpretation of weighted scores

**The weighting scheme emphasizes clinically consequential reasoning.** Core judgment and preferred management together account for 60% of the total score because errors in these domains are most likely to alter diagnosis, risk stratification, or treatment direction. Follow-up/referral planning and safety communication together account for 40% of the score because they determine whether management can be implemented safely and whether patients or caregivers understand warning signs, adherence requirements, and escalation pathways.

**The serious-safety-error flag complements the numerical score.** It was recorded separately to identify responses containing potentially unsafe clinical advice even when the aggregate score was not low. This allows the analysis to distinguish between incomplete but generally safe responses and responses with safety-critical failures.
